# Plasma Cell-free DNA Concentration and Outcomes from Taxane Therapy in Metastatic Castration-resistant Prostate Cancer from Two Phase III Trials (FIRSTANA and PROSELICA)

**DOI:** 10.1016/j.eururo.2018.02.013

**Published:** 2018-09

**Authors:** Niven Mehra, David Dolling, Semini Sumanasuriya, Rossitza Christova, Lorna Pope, Suzanne Carreira, George Seed, Wei Yuan, Jane Goodall, Emma Hall, Penny Flohr, Gunther Boysen, Diletta Bianchini, Oliver Sartor, Mario A. Eisenberger, Karim Fizazi, Stephane Oudard, Mustapha Chadjaa, Sandrine Macé, Johann S. de Bono

**Affiliations:** a Royal Marsden NHS Foundation Trust and The Institute of Cancer Research, London, UK; b The Institute of Cancer Research Clinical Trials and Statistics Unit, London, UK; c The Institute of Cancer Research, London, UK; d Tulane University School of Medicine, New Orleans, LA, USA; e The Sidney Kimmel Comprehensive Cancer Center at Johns Hopkins, Baltimore, MD, USA; f Department of Medical Oncology, Institut Gustave Roussy, University of Paris Sud, Villejuif, France; g Department of Medical Oncology, Hôpital Européen Georges Pompidou, Paris, France; h Sanofi, Paris, France

**Keywords:** Circulating cell-free DNA, cfDNA, Taxane chemotherapy, PROSELICA, FIRSTANA, Cabazitaxel, Docetaxel

## Abstract

**Background:**

Noninvasive biomarkers are needed to guide metastatic castration-resistant prostate cancer (mCRPC) treatment.

**Objective:**

To clinically qualify baseline and on-treatment cell-free DNA (cfDNA) concentrations as biomarkers of patient outcome following taxane chemotherapy.

**Design, setting, and participants:**

Blood for cfDNA analyses was prospectively collected from 571 mCRPC patients participating in two phase III clinical trials, FIRSTANA (NCT01308567) and PROSELICA (NCT01308580). Patients received docetaxel (75 mg/m^2^) or cabazitaxel (20 or 25 mg/m^2^) as first-line chemotherapy (FIRSTANA), and cabazitaxel (20 or 25 mg/m^2^) as second-line chemotherapy (PROSELICA).

**Outcome measurements and statistical analysis:**

Associations between cfDNA concentration and prostate-specific antigen (PSA) response were tested using logistic regression models. Survival was estimated using Kaplan-Meier methods for cfDNA concentration grouped by quartile. Cox proportional hazard models, within each study, tested for associations with radiological progression-free survival (rPFS) and overall survival (OS), with multivariable analyses adjusting for baseline prognostic variables. Two-stage individual patient meta-analysis combined results for cfDNA concentrations for both studies.

**Results and limitations:**

In 2502 samples, baseline log_10_ cfDNA concentration correlated with known prognostic factors, shorter rPFS (hazard ratio [HR] = 1.54; 95% confidence interval [CI]: 1.15–2.08; *p* = 0.004), and shorter OS on taxane therapy (HR = 1.53; 95% CI: 1.18–1.97; *p* = 0.001). In multivariable analyses, baseline cfDNA concentration was an independent prognostic variable for rPFS and OS in both first- and second-line chemotherapy settings. Patients with a PSA response experienced a decline in log_10_ cfDNA concentrations during the first four cycles of treatment (per cycle −0.03; 95% CI: −0.044 to −0.009; *p* = 0.003). Study limitations included the fact that blood sample collection was not mandated for all patients and the inability to specifically quantitate tumour-derived cfDNA fraction in cfDNA.

**Conclusions:**

We report that changes in cfDNA concentrations correlate with both rPFS and OS in patients receiving first- and second-line taxane therapy, and may serve as independent prognostic biomarkers of response to taxanes.

**Patient summary:**

In the past decade, several new therapies have been introduced for men diagnosed with metastatic prostate cancer. Although metastatic prostate cancer remains incurable, these novel agents have extended patient survival and improved their quality of life in comparison with the last decade. To further optimise treatment allocation and individualise patient care, better tests (biomarkers) are needed to guide the delivery of improved and more precise care. In this report, we assessed cfDNA in over 2500 blood samples from men with prostate cancer who were recruited to two separate international studies and received taxane chemotherapy. We quantified the concentration of cfDNA fragments in blood plasma, which partly originates from tumour. We identified that higher concentrations of circulating cfDNA fragments, prior to starting taxane chemotherapy, can be used to identify patients with aggressive prostate cancer. A decline in cfDNA concentration during the first 3–9 wk after initiation of taxane therapy was seen in patients deriving benefit from taxane chemotherapy. These results identified circulating cfDNA as a new biomarker of aggressive disease in metastatic prostate cancer and imply that the study of cfDNA has clinical utility, supporting further efforts to develop blood-based tests on this circulating tumour-derived DNA.

## Take Home Message

Plasma cell-free DNA comprises fragmented nucleic acids from normal and tumour cells, and has utility as a biomarker of radiological progression-free and overall survival from first- and second-line treatment with taxane in advanced prostate cancer.

## 1 Introduction

Prostate cancer (PCa) remains a major global healthcare challenge and lethal PCa remains a major cause of male cancer deaths [1]. Although most men with metastatic hormone-sensitive PCa respond well to androgen deprivation therapy (ADT) alone, their disease invariably recurs as metastatic castration-resistant prostate cancer (mCRPC). Docetaxel was introduced as a life-prolonging treatment for mCRPC in 2004, with taxanes gaining importance in the management of mCRPC [2], [3]. The TROPIC trial led to cabazitaxel being registered as a second-line taxane in 2010 [4]. Furthermore, the CHAARTED and STAMPEDE trials brought docetaxel to the hormone-sensitive setting in 2015, showing unprecedented survival benefit in combination with ADT for patients with metastatic disease [5], [6].

Other approved therapies for mCRPC include abiraterone, enzalutamide, radium-223, and sipuleucel-T; however, no optimal sequence of treatment or patient selection strategies have yet been established [7]. Currently, patients are assigned specific treatment types pragmatically, often with fitter patients prescribed chemotherapy, and less toxic drugs assigned earlier [8]. Identifying patients who are likely to benefit from specific treatment options remains a critically important unmet clinical need, and biomarkers predictive of early response to taxane therapy would help minimise overtreatment.

Potential use of cell-free DNA (cfDNA) as a prognostic and predictive biomarker of PCa, facilitating diagnosis and response to treatment, has been suggested [9], [10], [11]. In healthy volunteers, cfDNA levels are <5 ng/ml and reported to largely arise from haematopoietic cells [12]. Conversely, elevated cfDNA concentrations are present in the plasma of patients with PCa, where it comprises both circulating tumour DNA and normal DNA, with tumour content averaging 30%. Circulating tumour DNA has been reported to represent multiple tumour sites and is released through necrosis, apoptosis, and even active secretion [13], [14].

cfDNA is amenable to qualitative, for example, genetic and epigenetic, and quantitative analyses [13], [15], [16], [17]. In a study of patients with various advanced cancers, the median cfDNA concentration was 17 ng/ml, with the highest concentrations (53 ng/ml) seen in patients with mCRPC [18].

This substudy assessed the clinical utility of plasma cfDNA in patients with mCRPC who also received taxanes (docetaxel and cabazitaxel) in two phase III clinical trials (FIRSTANA [NCT01308567] and PROSELICA [NCT01308580]). We performed preplanned analyses of baseline and serial blood samples taken from 571 consenting patients, and investigated the prognostic value of baseline cfDNA concentration and whether changes in cfDNA concentration during the first 9 wk of taxane chemotherapy are associated with response.

## 2 Patients and methods

### 2.1 Patients

This study included patients participating in two prospective, randomised, open-label, international phase III trials: FIRSTANA (NCT01308567), evaluating superiority of cabazitaxel 20 mg/m^2^ (*n* = 389) or cabazitaxel 25 mg/m^2^ (*n* = 388) over docetaxel 75 mg/m^2^ (*n* = 391), each with 10 mg/d prednisone, as first-line chemotherapy for patients with mCRPC [19]; and PROSELICA (NCT01308580), a noninferiority study evaluating cabazitaxel 20 mg/m^2^ (*n* = 598) or 25 mg/m^2^ (*n* = 602) with 10 mg/d prednisone, as second-line therapy for patients with mCRPC who progressed on docetaxel [20]. These study designs are shown in Supplementary Figure 1. The primary end point of both studies was overall survival (OS). Secondary end points included radiological progression-free survival (rPFS), tumour response in patients with measurable disease, and prostate-specific antigen (PSA) response (≥50% response at week 12; ≥50% response at any time). As part of preplanned biomarker analysis, baseline and serial blood samples were collected from consenting patients for quantitative and qualitative evaluation of plasma cfDNA. Analyses of cfDNA were included in both PROSELICA and FIRSTANA study protocols; however, details of statistical analyses were not prespecified and are therefore to be considered exploratory.

### 2.2 Blood collection and cfDNA extraction

Blood was collected in heparinised plasma tubes (BD Vacutainer; BD Biosciences, San Jose, CA, USA) at screening, prior to commencing cycle 1 (C1; baseline), C2, and C4, and at the end of treatment. Baseline samples (screening and C1) were taken between 1 and 7 d apart. cfDNA was isolated from 1 ml of plasma using QIAamp Circulating Nucleic Acid kit (Qiagen, Hilden, Germany), as per the manufacturer's protocol. Of the 50 μl eluate, 10 μl was used for quantification in duplicates using the Quant-IT Picogreen HS DNA kit (ThermoFisher, Waltham, Massachussets, USA), utilising a BioTek microplate spectrophotometer at 480ex/520em.

### 2.3 Statistical analyses

Baseline characteristics of patients selected for the biomarker substudy were compared with those of patients not selected using χ^2^ test and *t*-test as indicated. Baseline characteristics were compared between trials using χ^2^ test and *t*-test as indicated. To evaluate the biological variability of cfDNA, the coefficient of variation was calculated from both screening and C1 baseline plasma log_10_ cfDNA concentration. For all subsequent analyses, the average of the two baseline samples was used, unless only a single baseline was available. Pearson's correlation (*r*) was tested for associations between baseline log_10_ cfDNA concentration and other continuous baseline prognostic variables. Median rPFS and OS were estimated using the Kaplan-Meier method, and multivariable Cox proportional hazard models tested for associations with rPFS and OS. The proportional hazards assumption was tested using Schoenfeld residuals, but not found to be violated for log_10_-transformed cfDNA concentration. Multivariable analyses were adjusted for the trial, randomised treatment, baseline Eastern Cooperative Oncology Group (ECOG) performance status, Gleason score, visceral disease, bone-only disease, pain at baseline, albumin, alkaline phosphatase (ALP), haemoglobin, lactate dehydrogenase (LDH), neutrophil-to-lymphocyte ratio (NLR), and PSA. Approximately 22% of patients were missing one or more of these variables, and these were considered to be missing at random. To ensure no loss in the efficiency of multivariable analyses, multiple imputation by chained equations with the above coefficients was used to generate 20 imputations and per imputation estimates were combined using Rubin's rules. The value of adding baseline cfDNA to models of rPFS and OS was assessed by calculating Uno's inverse-probability weighted C-index and receiver operating characteristic (ROC) area under the curve (AUC) values [21]. Bootstrapping was used to calculate the 95% confidence interval (CI) and the difference (delta) between C-indices of each of the models. Time-dependent incident dynamic ROC curves (with 19-mo OS and 10-mo rPFS end point, which represents the median survival of the dataset) and time-dependent AUC curves were calculated according to the method proposed by Blanche et al [22]. The change at C2 and C4 from baseline was calculated (ΔcfDNA). Associations between log_10_ cfDNA concentration, ΔcfDNA, and ΔcfDNA cut-off (>20% and >30% change) and PSA response were tested using per study logistic regression models. Cox models, utilising a landmark approach, assessed the association between these cfDNA values and OS and rPFS. Two-stage individual patient meta-analyses combined results for per-study logistic regression and Cox model analyses of average baseline log_10_ cfDNA concentration; results are displayed using forest plots. Linear mixed-effect models of log_10_ cfDNA concentrations during the first four cycles, with random patient intercept effects nested within random study intercept effects, assessed the association of patient characteristics. Characteristics included PSA response at any time, a PSA flare during the first 12 wk (defined as any increase followed by a 50% decline in PSA from baseline), and white blood cell (WBC) count at week 2. All *p* values <0.05 were considered significant. Stata v13, R v3.4.1, SPSS Statistics for Macintosh v22.0 (IBM Corp, Armonk, NY, USA), and GraphPad Prism version 6.0 for Macintosh (GraphPad Software, La Jolla, CA, USA) were used for statistical analyses and figures.

## 3 Results

### 3.1 Patients and samples

Overall, 571 patients with mCRPC (315 of 1168 patients enrolled in FIRSTANA and 256 of 1200 patients enrolled in PROSELICA) were included between April 2011 and December 2013 in this substudy evaluating the association of cfDNA concentration with outcomes from taxane chemotherapy. A total of 1400 patient samples from FIRSTANA and 1102 from PROSELICA were available. Patient baseline characteristics are presented in Table 1. Baseline characteristics of patients included in the biomarker substudy, compared with those who were not included, are presented in Supplementary Table 1. An imbalance in baseline characteristics was seen in FIRSTANA substudy patients for baseline pain, Gleason score, haemoglobin, albumin, and ALP. FIRSTANA included a docetaxel-naïve population, whereas PROSELICA included a post-docetaxel population with more advanced disease, with patients having higher ECOG performance status, LDH, ALP, and PSA levels and a lower haemoglobin concentration. In FIRSTANA, 203 patients died and 149 patients radiologically progressed, and in PROSELICA, 220 patients died and 142 patients radiologically progressed. The median follow-up periods for patients who did not die were 33 and 27mo for FIRSTANA and PROSELICA, respectively. The median follow-up periods for patients who did not experience radiological progression were 8 and 5 mo for FIRSTANA and PROSELICA, respectively. PSA responses, defined as confirmed ≥50% PSA falls by the Prostate Cancer Working Group 2 definition, were 68% and 43% in FIRSTANA and PROSELICA, respectively (*p* < 0.001).

**Table 1:** Baseline characteristics

Characteristic	FIRSTANA *n* = 315	PROSELICA *n* = 256	*p* value a
	*N* (%)	*N* (%)	
ECOG PS b			
0–1	305 (97)	235 (92)	0.008
2	10 (3)	21 (8)	
RECIST measurable b			
No	141 (45)	121 (47)	0.8
Yes	174 (55)	135 (53)	
Visceral disease			
No	245 (78)	183 (71)	0.08
Yes	70 (22)	73 (29)	
Pain at baseline c			
No	79 (28)	60 (26)	0.9
Yes	208 (72)	171 (74)	
Gleason score at diagnosis d			
<8	117 (39)	117 (49)	0.02
≥8	182 (61)	122 (51)	
Trial arm			
Cabazitaxel 20 mg/m^2^	111 (35)	120 (47)	<0.001
Cabazitaxel 25 mg/m^2^	89 (28)	136 (530)	
Docetaxel 75 mg/m^2^	115 (37)	0 (0)	

	Median (IQR)	Median (IQR)	*p* value e
Age (yr)	69 (63–74)	68 (64–73)	0.9
LDH (U/l)	234 (188–350)	331 (221–547)	<0.001
ALP (U/l)	113 (77–243)	178 (96–387)	<0.001
Haemoglobin (g/dl)	130.0 (119.2–137.0)	119.0 (106.0–127.5)	<0.001
Albumin (g/dl)	40.0 (37.5–43.0)	40.7 (37.0–43.1)	0.7
PSA (ng/ml)	80.0 (30.0–189.0)	207.6 (59.7–598.9)	<0.001
PSA doubling time (mo)	2.1 (1.3–3.4)	1.9 (1.3–3.1)	0.3
Log_10_ cfDNA concentration (ng/ml)	1.21 (0.97–1.54)	1.45 (1.18–1.86)	<0.001
NLR	3.0 (2.1–4.3)	3.7 (2.4–5.7)	<0.001

Outcome	*N* (%)	*N* (%)	*p* value a
>50% PSA response at 12 wk			
No	141 (46)	165 (69)	<0.001
Yes	163 (54)	73 (31)	
>50% PSA response at any time			
No	96 (32)	136 (57)	<0.001
Yes	208 (68)	102 (43)	

	Median (IQR)	Median (IQR)	HR (95% CI) *p* value f
rPFS (mo)	11.6 (7.9–18.2)	8.1 (4.2–12.7)	0.53 (0.42–0.68) <0.001
OS (mo)	25.6 (13.2–39.7)	14.4 (8.0–21.4)	0.42 (0.35–0.52) <0.001

ALP = alkaline phosphatase; cfDNA = cell-free DNA; CI = confidence interval; ECOG PS = Eastern Cooperative Oncology Group performance status; HR = hazard ratio; IQR = interquartile range; LDH = lactate dehydrogenase; NLR = neutrophil-lymphocyte ratio; OS = overall survival; PSA = prostate-specific antigen; RECIST = Response Evaluation Criteria in Solid Tumours; rPFS = radiological progression-free survival; U = unit.

a χ^2^ test.

b Stratification parameters.

c Fifty-three assessments missing (28 in FIRSTANA and 25 in PROSELICA).

d Thirty-three assessments missing (16 in FIRSTANA and 17 in PROSELICA).

e Wilcoxon rank-sum test.

f Proportional hazards Cox model.

### 3.2 Baseline cfDNA concentrations

**Fig. 1:**
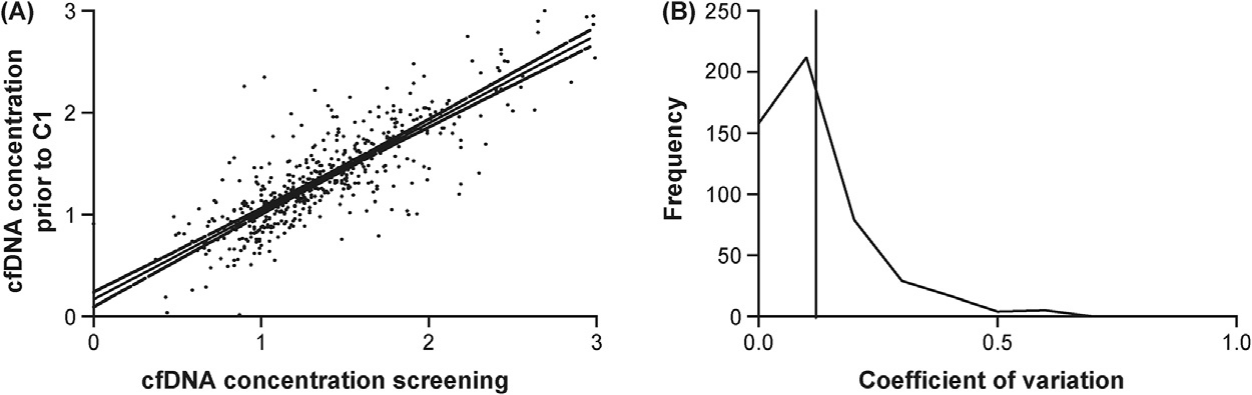
Correlation and coefficient of variation (CV) between both baseline samples taken between 1 and 7 d apart. (A) Relationship between log_10_-transformed cfDNA concentration (ng/ml) at screening and at C1 in 507 paired samples derived from *n* = 571 patients. Correlation coefficient = 0.84 (Pearson's rho) with *p* < 0.001. (B) CV of baseline samples depicted in a frequency chart with mean and median CV of 0.12 and 0.08 with 95% CI of 0.11 and 0.13, respectively. Mean CV is shown in solid line. C = cycle; cfDNA = cell-free DNA; CI = confidence interval; CV = coefficient of variation.

We first evaluated the biological variability in log_10_ cfDNA concentration between the two baseline samples, taken between 1 and 7 d apart, and collected in 507 (89%) patients. Both baseline sample concentrations correlated well (*r* = 0.84, *p* < 0.001), with a mean coefficient of variation between the biological replicate samples of 12% (95% CI: 11–13%; Fig. 1). There was a robust correlation between log_10_ cfDNA concentration and established prognostic variables [23], [24], including log_10_ LDH (*r* = 0.46, *p* < 0.001, *n* = 566), haemoglobin (*r* = −0.45, *p* < 0.001, *n* = 570), log_10_ ALP (*r* = 0.40, *p* < 0.001, *n* = 569), and log_10_ PSA (*r* = 0.34, *p* < 0.001, *n* = 568), with a weak association with white blood cells (*r* = 0.14, *p* = 0.001, *n* = 570) and albumin (*r* = −0.12, *p* = 0.004, *n* = 560). All prognostic variables and their relationship with baseline log_10_ cfDNA concentration in FIRSTANA and PROSELICA are presented in Supplementary Table 2, with ECOG performance status, presence of pain at baseline, ALP, haemoglobin concentration, LDH, PSA doubling time, and NLR associating significantly with cfDNA in both trials.

### 3.3 Baseline and longitudinal cfDNA concentrations and response to taxanes

Univariable logistic regression analyses indicated that baseline log_10_ cfDNA concentration did not associate with a confirmed PSA response in either FIRSTANA (odds ratio [OR] = 0.91; *p* = 0.7) or PROSELICA (OR = 0.76; *p* = 0.3), or in a two-stage meta-analysis combining results from both studies (OR = 0.82; *p* = 0.3; Supplementary Table 2). Similarly, there was no evidence that baseline log_10_ cfDNA concentration was associated with radiological response (Supplementary Fig. 2).

**Fig. 2:**
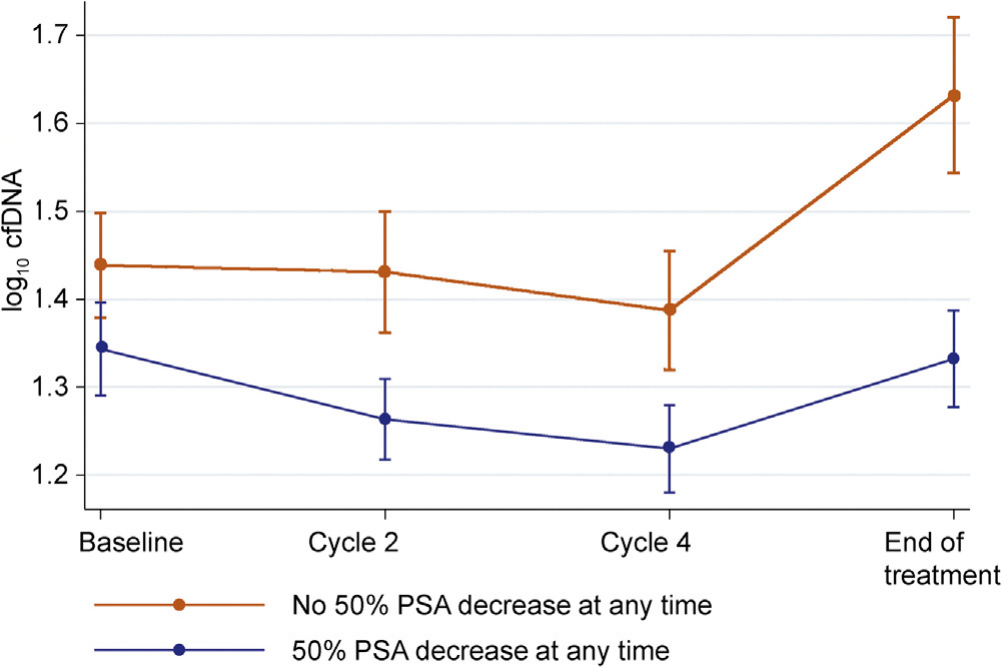
Mean log_10_ cfDNA concentrations and 95% confidence intervals per cycle by PSA response (decrease of 50% at any time). cfDNA = cell-free DNA; PSA = prostate-specific antigen.

We next evaluated the effect of taxane chemotherapy on longitudinal log_10_ cfDNA concentration during treatment. Mean plasma cfDNA concentration decreased during the first four cycles following chemotherapy initiation, consistent with taxane antitumour activity, but increased from baseline to the end of treatment in observance with disease progression. This trend was most obvious in the PROSELICA samples, already apparent at C2 following treatment initiation and reaching significance at C4 (Table 2). This is illustrated in Figure 2.

**Table 2:** Change in plasma cfDNA concentration (log_10_ ng/ml) from baseline in FIRSTANA and PROSELICA

	FIRSTANA			PROSELICA		
Change in Log_10_ plasma cfDNA concentration (ng/ml)	Mean change	95% CI	*n*	Mean change	95% CI	*n*
Cycle 2 (week 4)	−0.04	−0.08 to 0.007	280	−0.07	−0.12 to −0.02	231
Cycle 4 (week 10)	−0.04	−0.09 to 0.00	244	−0.07	−0.13 to −0.02	189
End of treatment	0.07	0.01–0.13	255	0.10	0.02–0.17	193

cfDNA = cell-free DNA; CI = confidence interval.

A multivariable mixed-effect model, displayed in Supplementary Table 3, analysed predictors of cfDNA concentrations during the first four cycles of treatment. There was no evidence of a difference in baseline cfDNA concentrations by PSA response (a 50% decline at any time) or of an overall per cycle change in cfDNA concentrations. Patients who had a PSA response (a 50% decline at any time) had lower per-cycle log_10_ cfDNA concentrations (−0.026; −0.044 to −0.009; *p* = 0.003) after adjusting for other baseline characteristics. There was no evidence that a PSA flare, experienced by 28/571 (4.9%) patients, influenced log_10_ cfDNA concentration.

Analyses of samples at C2 demonstrated that log_10_ cfDNA concentration, absolute change in log_10_ cfDNA concentration (at C2 compared with baseline or ΔcfDNA C2), and a >20% decline in log_10_ cfDNA concentration (at C2 compared with baseline) were associated with PSA response in PROSELICA. Analyses of samples at C4 (week 10) demonstrated that cfDNA parameters were significantly associated with PSA response in both studies; the absolute change in log_10_ cfDNA concentration (at C4 compared with baseline or ΔcfDNA C4) had an OR of 0.4 (95% CI: 0.2–0.7; *p* = 0.002) and 0.3 (95% CI: 0.2–0.6; *p* < 0.001) in FIRSTANA and PROSELICA, respectively, as well as in two-stage meta-analysis (OR 0.3; 95% CI: 0.2-0.5; *p* < 0.001). All other exploratory parameters and time points are given in Supplementary Table 4.

### 3.4 Radiological progression-free survival

**Fig. 3:**
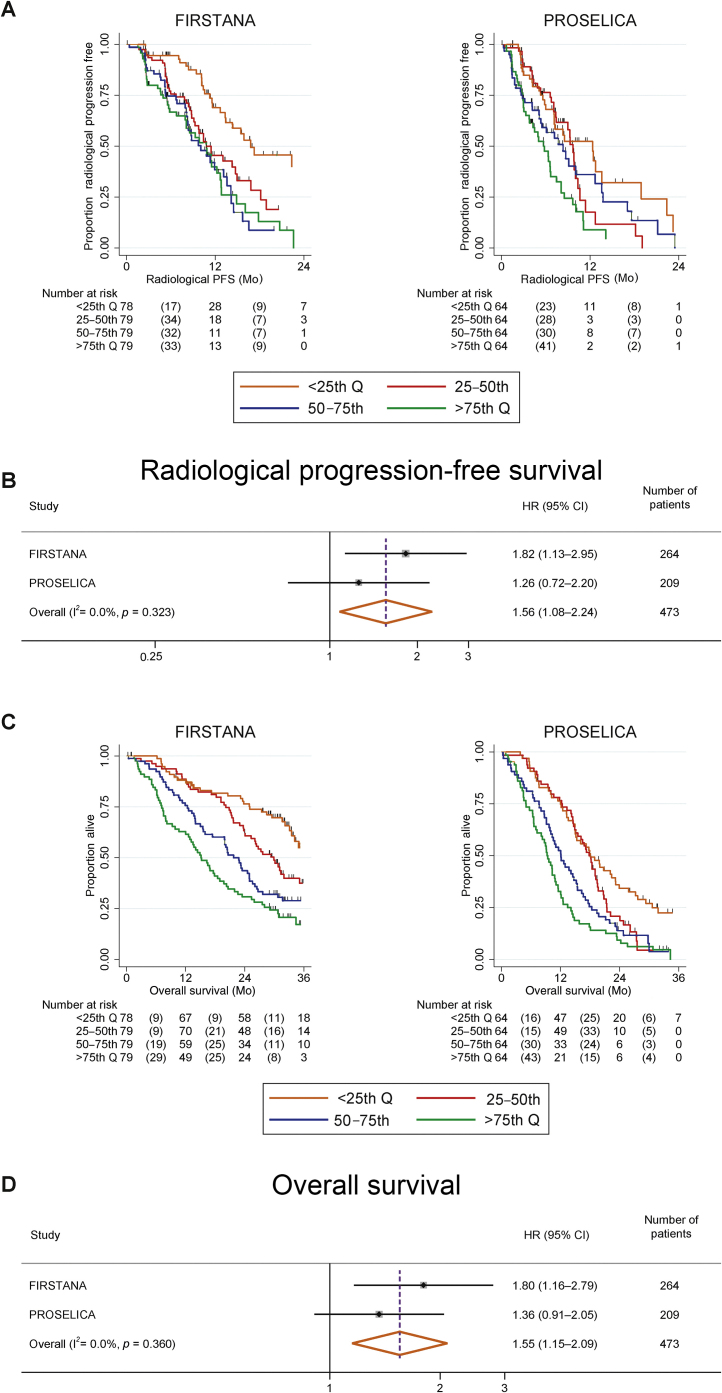
Correlation of baseline cfDNA concentration quartiles with rPFS and OS. (A) Kaplan-Meier curve of rPFS by baseline log_10_ cfDNA concentration quartiles. (B) Forest plot for rPFS (multivariable analysis model) for baseline log_10_ cfDNA concentration for each study and combined estimate. (C) Kaplan-Meier curve of OS by baseline cfDNA concentration quartiles. (D) Forest plot for OS (multivariable analysis model) for baseline log_10_ cfDNA concentration for each study and combined estimate. The multivariable Cox model included baseline log_10_ cfDNA concentration, ECOG PS at baseline (0 vs 1–2), visceral metastases, bone-only disease, Gleason score, baseline pain, baseline albumin, baseline ALP, baseline haemoglobin, baseline LDH, baseline NLR and baseline PSA. The *I*^2^ test displays and tests the level of heterogeneity between the studies, which is nonsignificant for cfDNA. ALP = alkaline phosphatase; cfDNA = cell-free DNA; CI = confidence interval; ECOG PS = Eastern Cooperative Oncology Group performance status; HR, = hazard ratio; LDH = lactate dehydrogenase; NLR = Neutrophil-lymphocyte ratio; OS = overall survival; PSA = prostate-specific antigen; rPFS = radiological progression-free survival.

Median rPFS was 17, 11, 10, and 11 mo in FIRSTANA, and 12, 10, 8, and 6 mo in PROSELICA for patients grouped by log_10_ cfDNA concentration from the lowest to the highest quartile (Fig. 3A). Multivariable survival analyses of baseline prognostic factors and rPFS for both studies combined are shown in Table 3; log_10_ cfDNA had an HR of 1.54 (95% CI: 1.15–2.08; *p* = 0.004). Uno's C-index for this model with time truncated at 24 mo was 0.70 (95% CI: 0.67–0.73), and this model did not provide significantly improved model fit compared with the model without log_10_ cfDNA (C-index: 0.69; 95% CI: 0.66–0.73; delta = 0.006; −0.003 to 0.02; *p* = 0.2). The area under the time-dependent ROC curve for the model with cfDNA at 10 mo was 0.78 (95% CI: 0.73–0.84) and was not significantly different from that for the model without cfDNA (*p* = 0.5; Supplementary Fig. 4). In both studies, post-treatment log_10_ cfDNA concentration at C2 and C4 was associated similarly with rPFS. Combined study estimates for log_10_ cfDNA concentration at C2 and C4 were HR = 1.89 (95% CI: 1.36–2.63; *p* < 0.001) and HR = 1.88 (95% CI: 1.32–2.68; *p* < 0.001), respectively. There was no evidence that the absolute change in log_10_ cfDNA concentration was associated with rPFS at C2 (HR = 1.12; 0.78–1.59; *p* = 0.5) or C4 (HR = 1.37; 95% CI: 0.92–2.02; *p* = 0.12) in the combined study estimates.

**Table 3:** Multivariable analyses of PFS and OS

Baseline characteristics	rPFS			OS		
	aHR	95% CI	*p* value	aHR	95% CI	*p* value
Log_10_ cfDNA	1.54	1.15–2.08	0.004	1.53	1.18–1.97	0.001
ECOG PS						
0–1	1.00	–	–	1.00	–	–
≥2	1.16	0.68–1.96	0.7	1.15	0.76–1.74	0.5
Visceral disease						
No	1.00	–	–	1.00	–	–
Yes	1.77	1.33–2.36	<0.001	1.46	1.15–1.86	0.002
Bone-only disease						
No	1.00	–	–	1.00	–	–
Yes	0.54	0.39–0.75	<0.001	0.79	0.62–1.01	0.06
Gleason score						
<8	1.00	–	–	1.00	–	–
≥8	1.43	1.11–1.85	0.006	1.17	0.95–1.44	0.13
Baseline pain						
No	1.00	–	–	1.00	–	–
Yes	1.20	0.88–1.63	0.3	1.29	1.00–1.67	0.06
Study						
FIRSTANA	1.00	–	–	1.00	–	–
PROSELICA	1.49	1.11–2.00	0.008	1.65	1.29–2.13	<0.001
Trial arm						
Cabazitaxel 20 mg/m^2^	1.00	–	0.8	1.00	–	0.6
Cabazitaxel 25 mg/m^2^	1.00	0.77–1.30	–	0.91	0.73–1.13	–
Docetaxel 75 mg/m^2^	1.14	0.79–1.64	–	1.04	0.77–1.41	–
LDH (Log_10_ U/l)	2.40	1.32–4.37	0.004	2.41	1.43–4.05	0.001
ALP (Log_10_ U/l)	1.12	0.78–1.61	0.5	1.53	1.14–2.04	0.004
Haemoglobin (g/dl)	0.85	0.77–0.94	<0.001	0.86	0.79–0.94	<0.001
Albumin (g/dl)	1.00	0.85–1.17	1	1.04	0.87–1.25	0.7
PSA (log_10_ ng/ml)	0.86	0.72–1.03	0.10	1.03	0.87–1.21	0.7
NLR (log_10_)	1.42	0.88–2.28	0.2	1.78	1.18–2.70	0.006

ALP = alkaline phosphatase; cfDNA = cell-free DNA; CI = confidence interval; ECOG PS = Eastern Cooperative Oncology Group performance status; aHR = adjusted hazard ratio; LDH = lactate dehydrogenase; NLR = neutrophil-lymphocyte ratio; OS = overall survival; PFS = progression-free survival; PSA = prostate-specific antigen; rPFS = radiological progression-free survival; U = unit.

### 3.5 Overall survival

Median OS for patients in FIRSTANA and PROSELICA was 39, 30, 22, and 15 mo, and 18, 18, 12, and 9 mo, respectively, for patients grouped by cfDNA concentration from the lowest to the highest quartile (Fig. 3C). Multivariable survival analyses of baseline prognostic factors and rPFS for both studies combined are shown in Table 3; log_10_ cfDNA had a HR of 1.53 (95% CI: 1.18–1.97; *p* = 0.001). Uno's C-index for this model with time truncated at 36 mo was 0.73 (95% CI: 0.70–0.75), although this model did not provide significantly improved model fit compared with the model without log_10_ cfDNA (C-index: 0.72; 95% CI: 0.70–0.75; delta = 0.004; −0.0009 to 0.008; *p* = 0.12). The area under the time-dependent ROC curve for the model with cfDNA at 19 mo was 0.82 (95% CI: 0.78–0.86) and was significantly higher than that for the model without cfDNA (*p* = 0.05; Supplementary Fig. 3). Comparing combined study–estimated OS of patients with post-treatment samples, results show that HR = 1.77 (95% CI: 1.37–2.29; *p* < 0.001) and HR = 1.75 (95% CI: 1.30–2.35; *p* < 0.001) at C2 and C4, respectively. There was no evidence of an association between the absolute change in log_10_ cfDNA concentration in the post-treatment samples and OS (C2 HR = 1.26; 95% CI: 0.94–1.68; *p* = 0.12; C4 HR = 1.29; 95% CI: 0.92–1.79; *p* = 0.14) in the combined study estimates.

## 4 Discussion

Quantitative assessment of plasma cfDNA levels can facilitate the diagnosis of PCa and predict biochemical recurrence following prostatectomy [9], [10], [11], [25], [26]. Previous small, mainly retrospective studies also indicated a relationship between baseline cfDNA concentration and OS [18], [27]. One of these studies evaluated 59 patients with mCRPC following first-line docetaxel or second-line cabazitaxel chemotherapy, suggesting a correlation between baseline median cfDNA concentration levels and extent of PSA decline [27]. A study of eight patients with mCRPC following docetaxel chemotherapy suggested a possible correlation between baseline cfDNA concentration and radiological response [28].

Our study, of 751 patients treated with taxane chemotherapy enrolled in two phase III trials, revealed that cfDNA concentration at baseline correlated with both rPFS and OS. Changes in cfDNA concentration during taxane treatment were associated with biochemical response, but baseline cfDNA levels showed no relationship with biochemical or radiological response. Notably, our analysis corrected for differences in prognostic variables, and between first- and second-line chemotherapy. This correction was not performed in the study by Kienel and colleagues [27], likely biasing the conclusions, since higher responses are observed in first-line patients who are more likely to have lower cfDNA concentrations. A study by Kwee and colleagues [28] reported an increase in cfDNA concentration following one and three cycles of docetaxel; in contrast, our analyses of cfDNA concentration showed distinctive kinetics of cfDNA following taxane chemotherapy between responders and nonresponders. Both FIRSTANA and PROSELICA studies observed a significant decline in cfDNA at week 10 in responding patients; in the second-line setting, a decline as early as week 4 after therapy was found to be associated with response. In summary, our data extend our knowledge on the prognostic value of cfDNA in patients with PCa. Limited additional value was seen following incorporation of cfDNA levels to prognostic models, and cfDNA level changes could not predict response to agent or dose level. In addition, changes in cfDNA following treatment did not meet surrogacy criteria of OS, defined by Prentice [29], as the effect of treatment on survival was not captured by this. Our study did not include internal validation, as we felt this was inappropriate due to differences in disease stages between study populations. Therefore, external validation is still warranted to confirm the clinical utility of quantitative cfDNA assessment as a prognostic biomarker.

There are limitations to cfDNA analyses in patient plasma. Plasma collection for cfDNA analyses in these two large trials was optional, resulting in only a proportion of patients having this collected. Baseline characteristics were not matched for recruited biomarker substudy patients in FIRSTANA; therefore, extrapolation of our results to the full dataset may only be made following correction for imbalances. Integrity of cfDNA may be compromised in the transportation, storage, and handling of samples; it has been shown that plasma cfDNA degrades by 30% for each year of storage [30], [31]. Other factors include high interpatient variability in cfDNA concentration. Although levels are generally found to be much higher in cancer patients than healthy volunteers, there is a significant degree of overlap, with a higher cfDNA concentration linked to inflammation as well as neoplasia [32]. Our results indeed imply that cfDNA constitutes both circulating tumour DNA and normal DNA, with haemoglobin, LDH, WBC, and PSA levels best explaining cfDNA levels. Changes in cfDNA levels were best explained by changes in tumour burden measures, LDH, and PSA decline. Of note, samples obtained from FIRSTANA and PROSELICA participants were stored for less than a year before cfDNA extraction, and the high concordance of biological replicates observed in this study suggests that compromised integrity was highly unlikely.

Another limitation to the interpretation of our data and the clinical utility of cfDNA as a prognostic biomarker is theoretically due to different proportions of tumour DNA constituting the total cfDNA concentration; this can vary significantly across tumour types, with circulating tumour DNA concentration between 0.01% and 95% [14], [15], [33]. Estimation of tumour content by bioinformatic algorithms incorporating information from single nucleotide polymorphisms and clonal mutations are evaluated in these samples, potentially increasing the utility of cfDNA as a biomarker.

## 5 Conclusions

Our study identifies baseline cfDNA concentration as an independent prognostic biomarker in patients with mCRPC, with higher baseline concentrations associated with shorter rPFS and OS following taxane therapy. A decline in total cfDNA concentration during the first 9 wk of treatment was associated with response to taxane therapy. This study is part of ongoing efforts to clinically qualify the utility of cfDNA in the management of advanced PCa patients.

  ***Author contributions:*** Johann S. de Bono had full access to all the data in the study and takes responsibility for the integrity of the data and the accuracy of the data analysis.  

*Study concept and design:* de Bono, Chadjaa, Macé.

*Acquisition of data:* Christova, Pope, Flohr, Goodall.

*Analysis and interpretation of data:* Mehra, Dolling, Sumanasuriya, Carreira, Seed, Yan, de Bono.

*Drafting of the manuscript:* Mehra, Dolling, Sumanasuriya, de Bono.

*Critical revision of the manuscript for important intellectual content:* Mehra, Dolling, Sumanasuriya, Carreira, Seed, Yuan, Goodall, Hall, Boysen, Bianchini, Sartor, Eisenberger, Fizazi, Oudard, Chadjaa, Macé, de Bono.

*Statistical analysis:* Mehra, Seed, Yan, Dolling, Hall.

*Obtaining funding:* None.

*Administrative, technical, or material support:* None.

*Supervision:* None.

*Other:* None.  

***Financial disclosures:*** Johann S. de Bono certifies that all conflicts of interest, including specific financial interests and relationships and affiliations relevant to the subject matter or materials discussed in the manuscript (eg, employment/affiliation, grants or funding, consultancies, honoraria, stock ownership or options, expert testimony, royalties, or patents filed, received, or pending), are the following: Rossitza Christova reports receiving a grant from Sanofi. Emma Hall reports grants to her institution from AstraZeneca, Bayer, Astellas, Sanofi, and Janssen. Oliver Sartor has provided a consultancy/advisory role for and received honoraria from Sanofi. Mario A. Eisenberger has provided a consultancy/advisory role for and received honoraria from Sanofi. Karim Fizazi has received honoraria from Janssen Oncology, Bayer, Astellas Pharma, Sanofi, Orion Pharma, and Amgen. Stephane Oudard has provided a consultancy/advisory role for and received honoraria from Sanofi, Ipsen, and Janssen Oncology, and reports a grant to his institution from Sanofi during the conduct of the study. Mustapha Chadjaa and Sandrine Macé are employees of Sanofi. Johann S. de Bono has provided a consultancy/advisory role for and received honoraria from Sanofi, AstraZeneca, GSK, Roche/Genentech, Merck, and Genmab. Niven Mehra, David Dolling, Semini Sumanasuriya, Lorna Pope, Suzanne Carreira, George Seed, Wei Yuan, Jane Goodall, Penny Flohr, Gunther Boysen, and Diletta Bianchini have no conflicts to report.  

***Funding/Support and role of the sponsor:*** This work was supported by Sanofi, which played a role in the design and conduct of the study; collection, management, preparation, and review of the data; and approval of the manuscript.  

***Acknowledgements:*** We thank all the clinical trial patients and their families. The biomarker work (cfDNA extraction, etc.) was (in part) funded by Sanofi. Editorial support was provided by Sharmin Bovill of MediTech Media, funded by Sanofi.
