# Supplementary material for: Plasma Cell-free DNA Concentration and Outcomes from Taxane Therapy in Metastatic Castration-resistant Prostate Cancer from Two Phase III Trials (FIRSTANA and PROSELICA)
Source: Eur Urol. 2018 Sep;74(3):283–91. doi: 10.1016/j.eururo.2018.02.013 (PMC6090941; doi:10.1016/j.eururo.2018.02.013)
Supplement: Supplementary file 1 [file mmc1.docx]

**Supplementary material**

**Supplementary Fig. 1 – FIRSTANA and PROSELICA study designs**


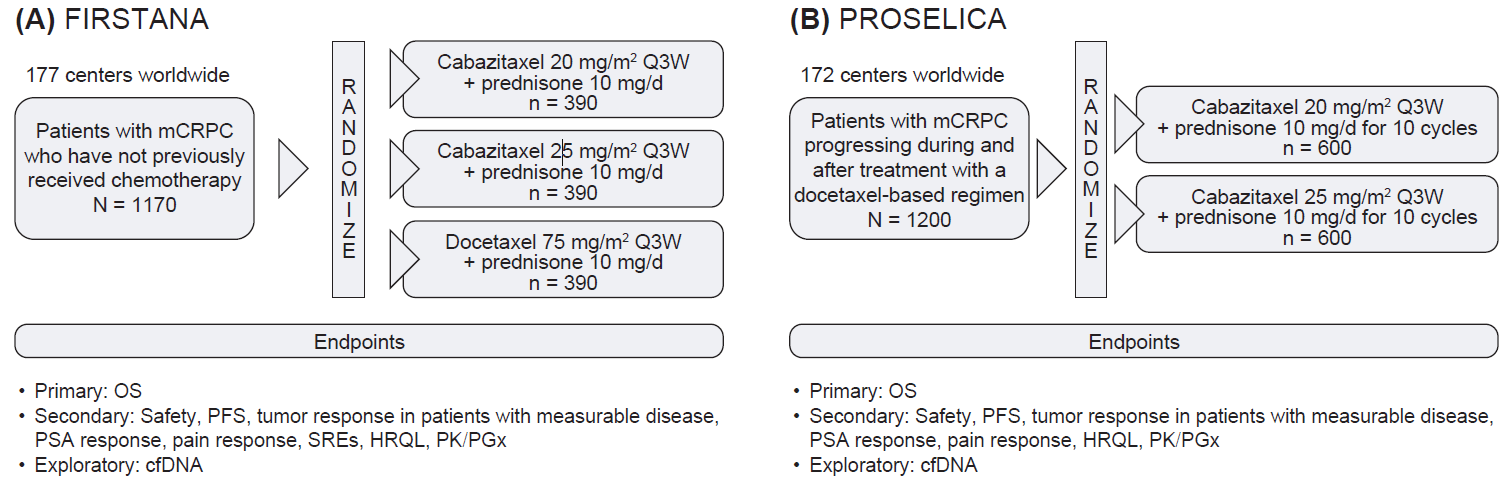


**Supplementary Fig. 2 – Multivariable logistic regression models of baseline log_10_ plasma cfDNA concentration correlation with (A) PSA response at Week 12 and (B) at any time, and with (C) radiological response**


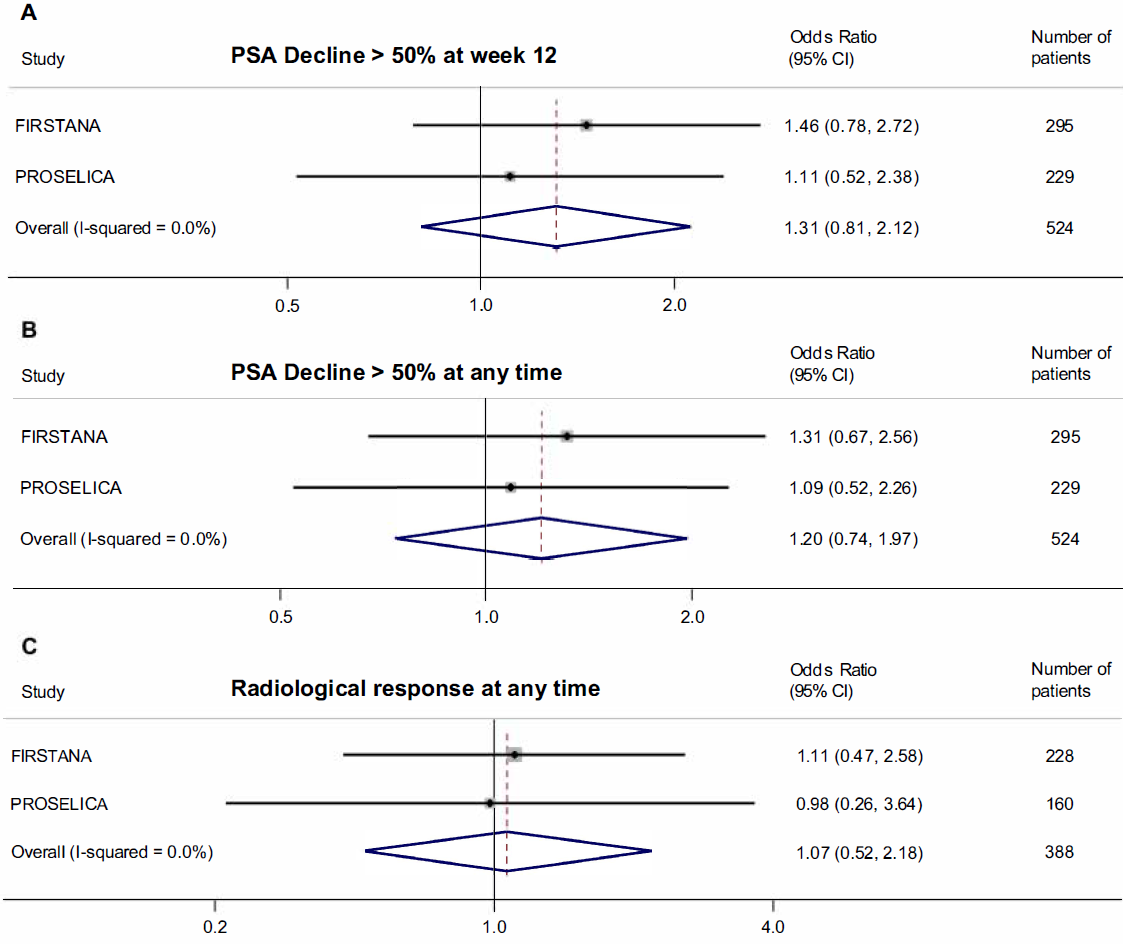


cfDNA = cell-free DNA; CI = confidence interval; OR = odds ratio; PSA = prostate-specific antigen.

**Supplementary Fig. 3 – ROC at 10 months (A) and time-dependent AUC for rPFS (B) and ROC (C) and AUC for OS (D)**


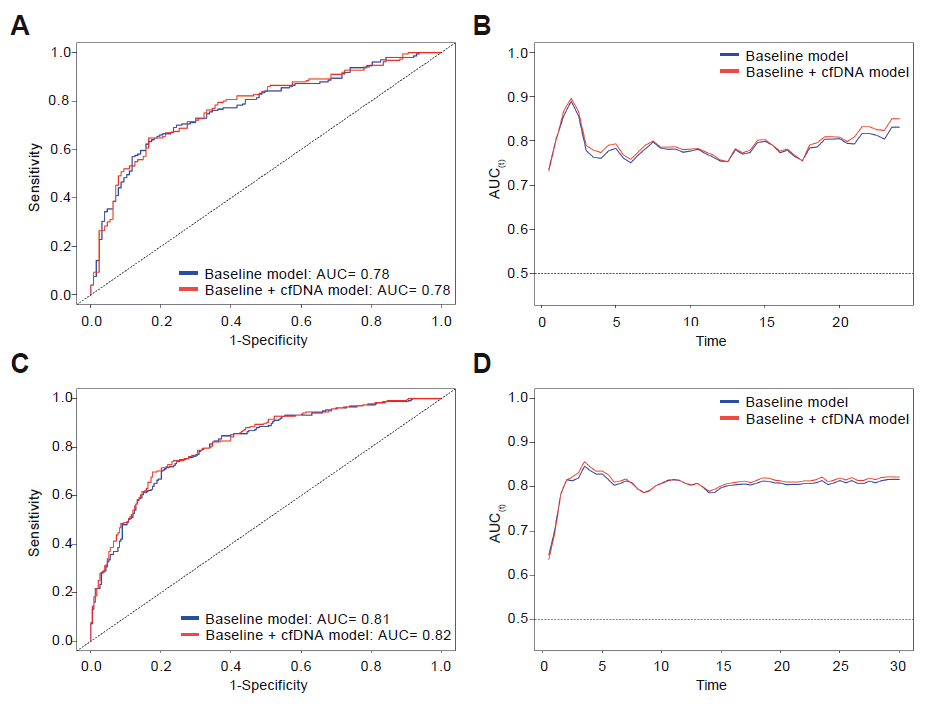


**Supplementary Table 1. Baseline characteristic comparison by biomarker subset status**

|  | **FIRSTANA** | | | **PROSELICA** | | |
| --- | --- | --- | --- | --- | --- | --- |
| **Characteristic** | **Biomarker subset**  **n=315** | **Non-biomarker subset**  **n=853** |  | **Biomarker subset**  **N=256** | **Non-biomarker subset**  **n=944** |  |
|  | **N (%)** | **N (%)** | ***p*-value** | **N (%)** | **N (%)** | ***p*-value** |
| ECOGPS |  |  |  |  |  |  |
| 0–1 | 305 (97) | 815 (96) | 0.33 | 235 (92) | 844 (89) | 0.26 |
| 2 | 10 (3) | 38 (4) |  | 21 (8) | 100 (11) |  |
| RECIST measurable |  |  |  |  |  |  |
| No | 141 (45) | 365 (43) | 0.48 | 121 (47) | 492 (52) | 0.14 |
| Yes | 174 (55) | 488 (57) |  | 135 (53) | 452 (48) |  |
| Visceral disease |  |  |  |  |  |  |
| No | 245 (78) | 673 (79) | 0.68 | 183 (71) | 682 (72) | 0.81 |
| Yes | 70 (22) | 180 (21) |  | 73 (29) | 262 (28) |  |
| Pain at baseline |  |  |  |  |  |  |
| No | 79 (28) | 295 (38) | 0.002 | 60 (26) | 250 (29) | 0.42 |
| Yes | 208 (72) | 487 (62) |  | 171 (74) | 622 (71) |  |
| Gleason score at diagnosis |  |  |  |  |  |  |
| <8 | 117 (39) | 374 (49) | *0.005* | 117 (49) | 420 (47) | 0.62 |
| ≥8 | 182 (61) | 395 (51) |  | 122 (51) | 471 (53) |  |
| Trial Arm |  |  |  |  |  |  |
| Cabazitaxel 20 mg/m^2^ | 111 (35) | 278 (33) | 0.09 | 120 (47) | 478 (51) | 0.29 |
| Cabazitaxel, 25mg/m^2^ | 89 (28) | 299 (35) |  | 136 (530) | 466 (49) |  |
| Docetaxel 75mg/m^2^ | 115 (37) | 276 (32) |  | 0 (0) |  |  |
|  | **Median (IQR)** | **Median (IQR)** | ***p*-value** | **Median (IQR)** | **Median (IQR)** | ***p*-value** |
| Age, years | 69 (63–74) | 68 (63–73) | 0.83 | 68 (64–73) | 69 (63–74) | 0.41 |
| LDH, U/L | 234 (188–350) | 244 (193–350) | 0.05 | 331 (221–547) | 324 (221–498) | 0.74 |
| ALP, U/L | 113 (77–243) | 131 (81–279) | 0.05 | 178 (96–387) | 163 (92–387) | 0.10 |
| Haemoglobin, g/dl | 130.0  (119.2–137.0) | 127.0 (116.0–136.0) | 0.02 | 119.0  (106.0–127.5) | 120 (109–130) | 0.12 |
| Albumin, g/dl | 40.0  (37.5–43.0) | 41.7 (38.0–44.5) | <0.001 | 40.7  (37.0–43.1) | 40.0  (36.6–43.0) | 0.17 |
| PSA, ng/ml | 80.0  (30.0–189.0) | 75.9 (28.9–201.3) | 0.77 | 207.6  (59.7–598.9) | 150.9 (53.3–394.1) | 0.02 |
| PSA-doubling time (months) | 2.1 (1.3–3.4) | 2.0 (1.3–3.3) | 0.95 | 1.9 (1.3–3.1) | 1.9 (1.2–3.1) | 0.78 |
| NLR | 3.0 (2.1–4.3) | 2.9 (2.0–4.1) | 0.14 | 3.7 (2.4–5.7) | 3.3 (2.2–5.4) | 0.06 |
| **Outcome** | **N (%)** | **N (%)** | ***p*-value** | **N (%)** | **N (%)** | ***p*-value** |
| >50% PSA response at 12 weeks |  |  |  |  |  |  |
| No | 148 (47) | 385 (45) | 0.57 | 178 (70) | 648 (69) | 0.79 |
| Yes | 167 (53) | 468 (55) |  | 78 (30) | 296 (31) |  |
| >50% PSA response at any time |  |  |  |  |  |  |
| No | 103 (33) | 242 (28) | 0.15 | 149 (58) | 562 (60) | 0.70 |
| Yes | 212 (67) | 611 (72) |  | 107 (42) | 382 (400 |  |
|  | **Median (IQR)** | **Median (IQR)** | **HR (95%CI)**  ***p*-value** | **Median (IQR)** | **Median (IQR)** | **HR (95%CI)**  ***p*-value** |
| rPFS, months | 11.6 (7.9–18.2) | 13.4 (8.0–24.1) | 1.17 (0.97–1.41)  0.10 | 8.1 (4.2–12.7) | 8.3 (4.1–12.4) | 0.97 (0.801.16) 0.72 |
| OS, months | 25.6  (13.2–39.7) | 24.3 (13.2–42.4) | 0.94 (0.80–1.11) 0.49 | 14.4  (8.0–21.4) | 14.0 (7.4–23.2) | 1.06 (0.91–1.23) 0.45 |

ALP = alkaline phosphatase; CI = confidence interval; ECOG PS = Eastern Cooperative Oncology Group performance status; HR = hazard ratio; IQR = interquartile range; LDH = lactate dehydrogenase; NLR = neutrophil-lymphocyte ratio; OS = overall survival; PSA = prostate-specific antigen; rPFS = radiographic progression-free survival.

**Supplementary Table 2. Prognostic factors associated with baseline cfDNA concentration in FIRSTANA and PROSELICA**

|  | **FIRSTANA** | | | | **PROSELICA** | | | |
| --- | --- | --- | --- | --- | --- | --- | --- | --- |
|  | **n** | **Mean log_10_ cfDNA** | **SD** | ***p*-value^[[1]](#footnote-1)^** | **n** | **Mean log_10_ cfDNA** | **SD** | ***p*-value^1^** |
| **Age** |  |  |  |  |  |  |  |  |
| < 65 | 106 | 1.21 | 0.40 | 0.04 | 76 | 1.58 | 0.52 | 0.30 |
| ≥ 65 | 209 | 1.32 | 0.46 |  | 180 | 1.51 | 0.47 |  |
| **ECOG PS at baseline** |  |  |  |  |  |  |  |  |
| 0 | 305 | 1.27 | 0.44 | 0.04 | 235 | 1.49 | 0.46 | <0.001 |
| 1–2 | 10 | 1.57 | 0.41 |  | 21 | 1.96 | 0.51 |  |
| **Prior radical treatment of the prostate** |  |  |  |  |  |  |  |  |
| No | 191 | 1.33 | 0.46 | 0.01 | 148 | 1.52 | 0.47 | 0.86 |
| Yes | 124 | 1.20 | 0.40 |  | 108 | 1.54 | 0.51 |  |
| **Bone-only disease** |  |  |  |  |  |  |  |  |
| No | 240 | 1.29 | 0.45 | 0.30 | 179 | 1.55 | 0.50 | 0.40 |
| Yes | 75 | 1.23 | 0.43 |  | 77 | 1.49 | 0.44 |  |
| **Nodal-only disease** |  |  |  |  |  |  |  |  |
| No | 299 | 1.29 | 0.45 | 0.06 | 246 | 1.54 | 0.49 | 0.009 |
| Yes | 16 | 1.07 | 0.30 |  | 10 | 1.14 | 0.25 |  |
| **Visceral metastases** |  |  |  |  |  |  |  |  |
| No | 245 | 1.26 | 0.47 | 0.16 | 183 | 1.50 | 0.47 | 0.08 |
| Yes | 70 | 1.35 | 0.33 |  | 73 | 1.61 | 0.52 |  |
| **Pain at baseline** |  |  |  |  |  |  |  |  |
| No | 79 | 1.18 | 0.37 | 0.04 | 70 | 1.36 | 0.34 | <0.001 |
| Yes | 208 | 1.30 | 0.44 |  | 171 | 1.61 | 0.52 |  |
| **Gleason score at baseline** |  |  |  |  |  |  |  |  |
| <8 | 117 | 1.24 | 0.48 | 0.24 | 117 | 1.57 | 0.49 | 0.40 |
| ≥8 | 182 | 1.31 | 0.42 |  | 122 | 1.52 | 0.48 |  |
| **Albumin** |  |  |  |  |  |  |  |  |
| < 35 g/L | 29 | 1.46 | 0.42 | 0.02 | 36 | 1.67 | 0.50 | 0.08 |
| ≥ 35 g/L | 280 | 1.26 | 0.44 |  | 215 | 1.51 | 0.48 |  |
| **ALP** |  |  |  |  |  |  |  |  |
| ≤ ULN | 153 | 1.17 | 0.37 | <0.001 | 79 | 1.33 | 0.38 | <0.001 |
| > ULN | 156 | 1.39 | 0.48 |  | 172 | 1.63 | 0.50 |  |
| **Haemoglobin** |  |  |  |  |  |  |  |  |
| < LLN | 163 | 1.42 | 0.49 | <0.001 | 206 | 1.57 | 0.49 | 0.005 |
| ≥ LLN | 151 | 1.13 | 0.33 |  | 50 | 1.36 | 0.41 |  |
| **LDH** |  |  |  |  |  |  |  |  |
| ≤ ULN | 85 | 1.13 | 0.30 | 0.001 | 36 | 1.22 | 0.30 | <0.001 |
| > ULN | 227 | 1.34 | 0.47 |  | 218 | 1.58 | 0.49 |  |
| **PSA doubling time** |  |  |  |  |  |  |  |  |
| < 2 months | 137 | 1.34 | 0.48 | 0.01 | 124 | 1.62 | 0.49 | 0.002 |
| ≥ 2 months | 151 | 1.22 | 0.40 |  | 108 | 1.42 | 0.44 |  |
| **NLR at baseline** |  |  |  |  |  |  |  |  |
| <3 | 157 | 1.20 | 0.40 | <0.001 | 88 | 1.42 | 0.48 | 0.01 |
| ≥3 | 156 | 1.36 | 0.47 |  | 167 | 1.58 | 0.48 |  |
| **Trial Arm** |  |  |  |  |  |  |  |  |
| Cabazitaxel 20 mg/m^2^ | 111 | 1.27 | 0.49 |  | 120 | 1.53 | 0.49 | 0.96 |
| Cabazitaxel 25mg/m^2^ | 89 | 1.22 | 0.42 | 0.19^*^ | 136 | 1.53 | 0.49 |  |
| Docetaxel 75mg/m^2^ | 115 | 1.34 | 0.40 |  | NA | - | - | - |

ALP = alkaline phosphatase; cfDNA = cell-free DNA; ECOG PS = Eastern Cooperative Oncology Group performance status; LDH = lactate dehydrogenase; PSA = prostate-specific antigen; SD = standard deviation; ULN = upper limit of normal.

*ANOVA.

**Supplementary Table 3: Multivariable mixed effect model of cfDNA during the first four cycles of treatment.**

| **cfDNA (log_10_)** | **Coef.** | **95% CI** | **p-value** |
| --- | --- | --- | --- |
| **Cycle** | 0.001 | -0.012 to 0.015 | 0.841 |
| **PSA decrease of 50% at any time** | -0.017 | -0.084 to 0.051 | 0.631 |
| **PSA decrease of 50% at any time#Cycle^[[2]](#footnote-2)^** | -0.026 | -0.044 to -0.009 | 0.003 |
| **ECOG PS** |  |  |  |
| 0–1 | 0.000 | - | - |
| 2 | 0.215 | 0.083 to 0.346 | 0.001 |
| **Trial Arm** |  |  |  |
| Cabazitaxel 20 mg/m^2^ | 0.000 | - | 0.974 |
| Cabazitaxel, 25mg/m^2^ | -0.005 | -0.069 to 0.06 | - |
| Docetaxel, 75mg/m^2^ | -0.008 | -0.087 to 0.071 | - |
| **Gleason score at diagnosis** |  |  |  |
| <8 | 0.000 | - | - |
| ≥8 | 0.057 | -0.002 to 0.117 | 0.058 |
| **Visceral disease** | 0.047 | -0.024 to 0.118 | 0.198 |
| **Bone-only disease** | -0.011 | -0.08 to 0.058 | 0.757 |
| **Baseline Pain** | 0.030 | -0.039 to 0.098 | 0.393 |
| **Albumin (g/dl)** | 0.004 | -0.043 to 0.051 | 0.858 |
| **ALP (log_10_ U/L)** | 0.077 | -0.011 to 0.164 | 0.086 |
| **Haemoglobin (g/dl)** | -0.056 | -0.079 to -0.033 | <0.001 |
| **LDH (log_10_ U/L)** | 0.485 | 0.336 to 0.634 | <0.001 |
| **NLR (log_10_)** | 0.089 | -0.026 to 0.204 | 0.128 |
| **PSA (log_10_ ng/ml)** | 0.080 | 0.035 to 0.124 | 0.001 |
| **PSA flare (any increase from baseline followed by 50% decrease)** | 0.107 | -0.022 to 0.236 | 0.105 |
| **WBC (Week 2)** | 0.015 | 0.007 to 0.023 | <0.001 |
| **Constant** | 0.274 | -0.279 to 0.826 | 0.331 |

ALP = alkaline phosphatase; cfDNA = cell-free DNA; ECOG PS = Eastern Cooperative Oncology Group performance status; LDH = lactate dehydrogenase; NLR = neutrophil lymphocyte ratio; PSA = prostate-specific antigen; WBC = white blood cell count.

**Supplementary Table 4. Univariate logistic regression of >50% decline in PSA at any time by cfDNA concentrations**

|  | **FIRSTANA** | | | **PROSELICA** | | | **OVERALL** | | |
| --- | --- | --- | --- | --- | --- | --- | --- | --- | --- |
|  | **OR** | **95% CI** | ***p*-value** | **OR** | **95% CI** | ***p*-value** | **OR** | **95%CI** | ***p*-value** |
| **Log_10_ cfDNA concentrationbaseline** | 0.91 | 0.53–1.56 | 0.73 | 0.76 | 0.44–1.31 | 0.32 | 0.82 | 0.56–1.20 | 0.30 |
| **Log_10_ cfDNA concentration C2** | 0.73 | 0.40–1.34 | 0.32 | 0.37 | 0.20–0.69 | **0.002** | 0.51 | 0.33–0.78 | **0.002** |
| **Log_10_ cfDNA concentrationC4** | 0.38 | 0.20–0.69 | **0.002** | 0.42 | 0.24–0.71 | **0.001** | 0.39 | 0.26–0.59 | **<0.001** |
| **Change in log_10_ cfDNA concentration** |  |  |  |  |  |  |  |  |  |
| C2 | 0.71 | 0.36–1.40 | 0.32 | 0.43 | 0.21–0.90 | **0.02** | 0.56 | 0.34–0.92 | **0.02** |
| C4 | 0.37 | 0.20–0.69 | **0.002** | 0.32 | 0.17–0.59 | **<0.001** | 0.34 | 0.22–0.53 | **<0.001** |
| **Log_10_ cfDNA concentrationdecline >20%** |  |  |  |  |  |  |  |  |  |
| C2 | 1.72 | 0.87–3.39 | 0.12 | 1.80 | 0.95–3.40 | 0.07 | 1.76 | 1.10–2.79 | **0.02** |
| C4 | 6.57 | 1.96–22.00 | **0.002** | 3.56 | 1.66–7.61 | **0.001** | 4.29 | 2.30–8.01 | **<0.001** |
| **Log_10_ cfDNA concentrationdecline >30%** |  |  |  |  |  |  |  |  |  |
| C2 | 2.31 | 0.92–5.80 | 0.07 | 1.64 | 0.73–3.69 | 0.23 | 1.92 | 1.05–3.47 | **0.03** |
| C4 | 9.38 | 1.24–71.08 | 0.03 | 1.81 | 0.72–4.53 | 0.21 | 2.78 | 1.30–5.94 | **0.009** |
| **Log_10_ cfDNA concentrationincrease >20%** |  |  |  |  |  |  |  |  |  |
| C2 | 1.37 | 0.71–2.62 | 0.35 | 0.46 | 0.22–0.97 | **0.04** | 0.85 | 0.54–1.35 | 0.50 |
| C4 | 0.93 | 0.51–1.71 | 0.83 | 0.53 | 0.26–1.09 | 0.09 | 0.74 | 0.47–1.17 | 0.20 |
| **Log_10_ cfDNA concentration increase >30%** |  |  |  |  |  |  |  |  |  |
| C2 | 1.22 | 0.58–2.59 | 0.59 | 0.47 | 0.19–1.19 | 0.11 | 0.84 | 0.49–1.45 | 0.54 |
| C4 | 0.80 | 0.42–1.53 | 0.50 | 0.49 | 0.21–1.13 | 0.09 | 0.67 | 0.41–1.11 | 0.12 |

C = cycle; cfDNA = cell-free DNA; CI = confidence interval; OR = odds ratio; PSA = prostate-specific antigen.

1. t-test [↑](#footnote-ref-1)
2. # denotes an interaction term [↑](#footnote-ref-2)
